# The absence of an invasive air sac system in the earliest dinosaurs suggests multiple origins of vertebral pneumaticity

**DOI:** 10.1038/s41598-022-25067-8

**Published:** 2022-12-09

**Authors:** Tito Aureliano, Aline M. Ghilardi, Rodrigo T. Müller, Leonardo Kerber, Flávio A. Pretto, Marcelo A. Fernandes, Fresia Ricardi-Branco, Mathew J. Wedel

**Affiliations:** 1 Institute of Geosciences, University of Campinas (Unicamp), Campinas, Brazil; 2 Diversity, Ichnology and Osteohistology Laboratory (DINOlab), Department of Geology, Federal University of Rio Grande Do Norte (URFN), Natal, Brazil; 3 Centro de Apoio À Pesquisa Paleontológica da Quarta Colônia (CAPPA), Federal University of Santa Maria (CAPPA/UFSM), São João Do Polêsine, Brazil; 4 Programa de Pós-Graduação Em Biodiversidade Animal, Federal University of Santa Maria (UFSM), Santa Maria, Brazil; 5 Laboratório de Paleoecologia E Paleoicnologia (LPP), Departamento de Ecologia E Biologia Evolutiva (DEBE), Federal University of São Carlos (UFSCar), São Carlos, Brazil; 6 College of Osteopathic Medicine of the Pacific and College of Podiatric Medicine, Western University of Health Sciences, Pomona, USA

**Keywords:** Palaeontology, Bone, Respiration

## Abstract

The origin of the air sac system present in birds has been an enigma for decades. Skeletal pneumaticity related to an air sac system is present in both derived non-avian dinosaurs and pterosaurs. But the question remained open whether this was a shared trait present in the common avemetatarsalian ancestor. We analyzed three taxa from the Late Triassic of South Brazil, which are some of the oldest representatives of this clade (233.23 ± 0.73 Ma), including two sauropodomorphs and one herrerasaurid. All three taxa present shallow lateral fossae in the centra of their presacral vertebrae. Foramina are present in many of the fossae but at diminutive sizes consistent with neurovascular rather than pneumatic origin. Micro-tomography reveals a chaotic architecture of dense apneumatic bone tissue in all three taxa. The early sauropodomorphs showed more complex vascularity, which possibly served as the framework for the future camerate and camellate pneumatic structures of more derived saurischians. Finally, the evidence of the absence of postcranial skeletal pneumaticity in the oldest dinosaurs contradicts the homology hypothesis for an invasive diverticula system and suggests that this trait evolved independently at least 3 times in pterosaurs, theropods, and sauropodomorphs.

## Introduction

One of the key features that granted the successful evolution and diversification of birds is the postcranial skeletal pneumaticity (PSP) associated with an air sac system, allowing the derivation of lightweight hyperventilated bodies capable of flight^1–3^. Of particular importance are the pneumatic diverticula, air-filled epithelial protrusions of the lungs and respiratory air sacs. These diverticula permeate the body, leaving several traces of their interaction with the skeleton^4–6^. Such traces have been described in fossil taxa in three distinct avemetatarsalian clades: pterosaurs^7,8^, sauropods^9–11^ and theropods^12–14^. A crucial question is whether this trait is homologous to ornithodirans, or developed independently two or three times in this group^15–17^. O’Connor^3^ proposed a method to evaluate unambiguous evidence of PSP in the fossil record. These three rules consist in observing three lines of evidence: the presence of well-developed fossae and laminae; the evidence of foramina inside these fossae; and the connection of these foramina with internal pneumatic structures (e.g. chambers, camerae, camellae). Novel computed tomography technologies and new key fossils from South America now allow us to address this long-standing enigma. A recently-described dinosaur fauna from the Late Triassic (early Carnian Candelária Sequence, Santa Maria Formation) of South Brazil accounts for the oldest dated taxa of this clade (233.23 ± 0.73^18^). In this study, we analyzed the axial skeletons of the early diverging sauropodomorphs *Buriolestes*^19^ and *Pampadromaeus*^20^, and the herrerasaurid *Gnathovorax*^21^ to evaluate the presence of unambiguous PSP and discuss the evolution of PSP in avemetatarsalians.

## Materials and method

### Institutional abbreviations

CAPPA/UFSM, Centro de Apoio à Pesquisa Paleontológica da Quarta Colônia, Universidade Federal de Santa Maria, São João do Polêsine, Rio Grande do Sul, Brazil; LPP-PV, Laboratório de Paleoecologia e Paleoicnologia (UFSCar), Federal University of São Carlos (UFSCar), São Carlos, São Paulo, Brazil; OMNH, Oklahoma Museum of Natural History, Norman, Oklahoma, United States of America; PVL, Paleovertebrate collection, Instituto “Miguel Lillo”, San Miguel de Tucumán, Salta, Argentina; MPCA, Museo Municipal de Ciencias Naturales “Carlos Ameghino”, Mercedes, Buenos Aires, Argentina; MOR, Museum of the Rockies, Bozeman, Montana, United States of America; NHM, Natural History Museum, London, United Kingdom; ULBRA, Centro de Apoio à Pesquisa Paleontológica da Quarta Colônia, Universidade Federal de Santa Maria, São João do Polêsine, Rio Grande do Sul, Brazil (previously Museu de Ciências Naturais, Universidade Luterana do Brasil, Canoas, Brazil).

### Specimens

The studied specimen of *Buriolestes schultzi* (CAPPA/UFSM 0035) corresponds to a nearly complete articulated skeleton and one of the best-known early dinosaurs^19^. It is one of the oldest sauropodomorphs and the basalmost taxon within this clade (but see^22^). The holotype of *Gnathovorax cabreirai* (CAPPA/UFSM-0009) corresponds to a nearly complete articulated skeleton, with some dorsal axial segments still under preparation^21^. It is one of the oldest and best-preserved herrerasaurids ever found to date. The holotype of *Pampadromaeus barberenai* (ULBRA-PV016) is an unarticulated almost complete skeleton^20^. Since the ULBRA collection was closed, ULBRA-PV016 is now housed in CAPPA/UFSM. Whereas histological thin sections were not performed, some structures demonstrate that all specimens reached an advanced degree of skeletal maturity, indicating that they were not juveniles at the time of death. Such evidence is based on the neurocentral sutures, the presence and shape of some muscle attachment structures, such as the femoral trochanteric shelf and anterolateral scar in the proximal portion of the femur, and dorsolateral trochanter^23–25^.

### Locality and horizon

**Figure 1:**
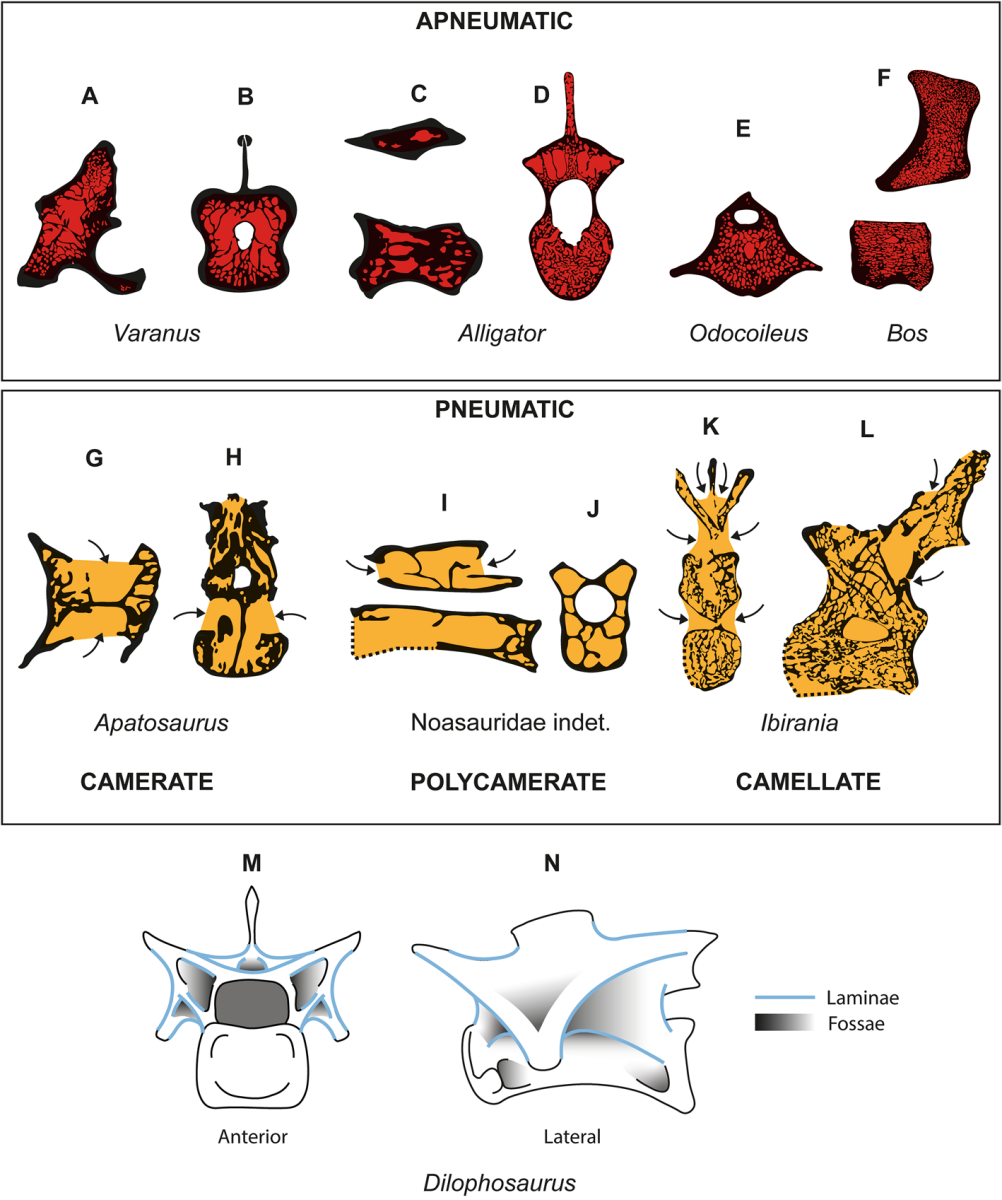
Examples of vertebrae with apneumatic (**A**–**F**) and pneumatic (**G**–**L**) architectures, and schematic drawings of vertebral fossae and laminae in (**M** and **N**). (**A**,**B**) *Varanus komodoensis* (NHMUK RR 1934.9.2.2)^15^. (**C**,**D**) *Alligator mississippiensis* (OUVC 11415 and NHMUK RR 1975.14.23, respectively). (**E**) *Odocoeileus virginianus*. (**F**) *Bos taurus*. (**G**,**H**) *Apatosaurus* sp. (MOR 957 6-29-92)^35^. (**I**,**J**) noasauridae indet. (DGM 929-R)^36^. (**K,L**) *Ibirania parva* (LPP-PV-0200)^37^. (**M**,**N**) *Dilophosaurus wetherilli*^38^. Lateral view in (**A**,**C**,**F**,**I**,**L**,**N**). Transverse sections in (**B**,**D**,**E**,**J**,**K**,**M**). Ventral view in (**G**). High contrast outlines in A-D and G-L based on CT scan data. (**M** and **N**) are based on the original schematic drawing of Adam Marsh^38^. WitmerLab at Ohio University provided access to the *Alligator* data originally appearing on their website, the collection of which was funded by NSF. The file was downloaded from www.MorphoSource.org, Duke University. Not to scale. Figures were generated with Adobe Illustrator CC version 22 X64.

*Buriolestes* was excavated at the Buriol Site (29°39′34.2″ S, 53°25′47.4″ W) and *Gnathovorax* at the Marchezan Site (29°37′52′′ S, 53°27′02′′ W), both at São João do Polêsine municipality, Rio Grande do Sul state, South Brazil. These sites are part of the Alemoa Member, Upper Triassic (Carnian) Santa Maria Formation, Paraná Basin. Their silty sandstone beds are included in the *Hyperodapedon* Assemblage Zone within the Acme Zone^26^, and absolute zircon dating for these beds points to 233.23 ± 0.73 Ma^18^. *Pampadromaeus* was collected at the “Várzea do Agudo” (= Janner) Site (29°39′10.89″ S, 53°17′34.20″ W), Agudo municipality, also Rio Grande do Sul state. The context is also the Alemoa Member, *Hyperodapedon* Assemblage, but at the immediately overlaying *Exaeretodon* Sub-zone stratum^20^. Therefore, it is known that *Pampadromaeus* pertained to a faunal assemblage slightly more recent than that of *Buriolestes* and *Gnathovorax* (see Fig. 1).

### Taphonomic remarks

CAPPA/UFSM 0035 and 0009 were not significantly affected by taphonomic compression, whereas several bone elements of ULBRA-PV016 are diagenetically distorted, not reflecting their natural shape. Nevertheless, the external texture of the elements of ULBRA-PV016, as well as that of the other two specimens, is well-preserved, depicting fine details of its anatomy. Opaque diagenetic minerals are also present in CAPPA/UFSM 0035 and 0009 as well as moderate calcite permineralization.

### Anatomical nomenclature

Wilson’s nomenclature was applied for vertebral laminae and fossae^6,27,28^ and Wedel’s terminology for vertebral pneumatic structures^5,29,30^. We followed O’Connor’s method to identify unambiguous evidence of PSP in our specimens^3^. Figure 1 exemplifies vertebrae of extinct and living taxa, and indicates structures such as laminae and fossae, illustrating different types of pneumatic and apneumatic architectures.

### Micro-computed tomography (μCT scan)

The anterior (third) cervical vertebra and an articulated posterior cervical vertebra of *Gnathovorax* were scanned. Dorsals are still under preparation in the jacket and were not available for this analysis. Almost the entire cervical series of *Buriolestes* was scanned as well as two middle dorsals. Finally, the three available dorsal vertebrae of *Pampadromaeus* were scanned.

Specimens were scanned on a Bruker-Skyscan 1173 microtomographer equipped with a 130-kV μ-focus X-ray source with a voxel size of 0.15 mm. The procedure took place at the *Instituto do Petróleo e dos Recursos Naturais* at the Universidade Católica do Rio Grande do Sul/PUCRS), Porto Alegre (Brazil). *3D-Slicer* v5.2^31^ and *CloudCompare* v2.9.1^32^ were used to analyze the data by applying color grades based on bone tissue density^33^. Digital measurements were taken with *ImageJ* v1.52^34^. All microtomography data was uploaded to the Morphobank platform and is available through this link: http://morphobank.org/permalink/?P4477.

## Results

**Figure 2:**
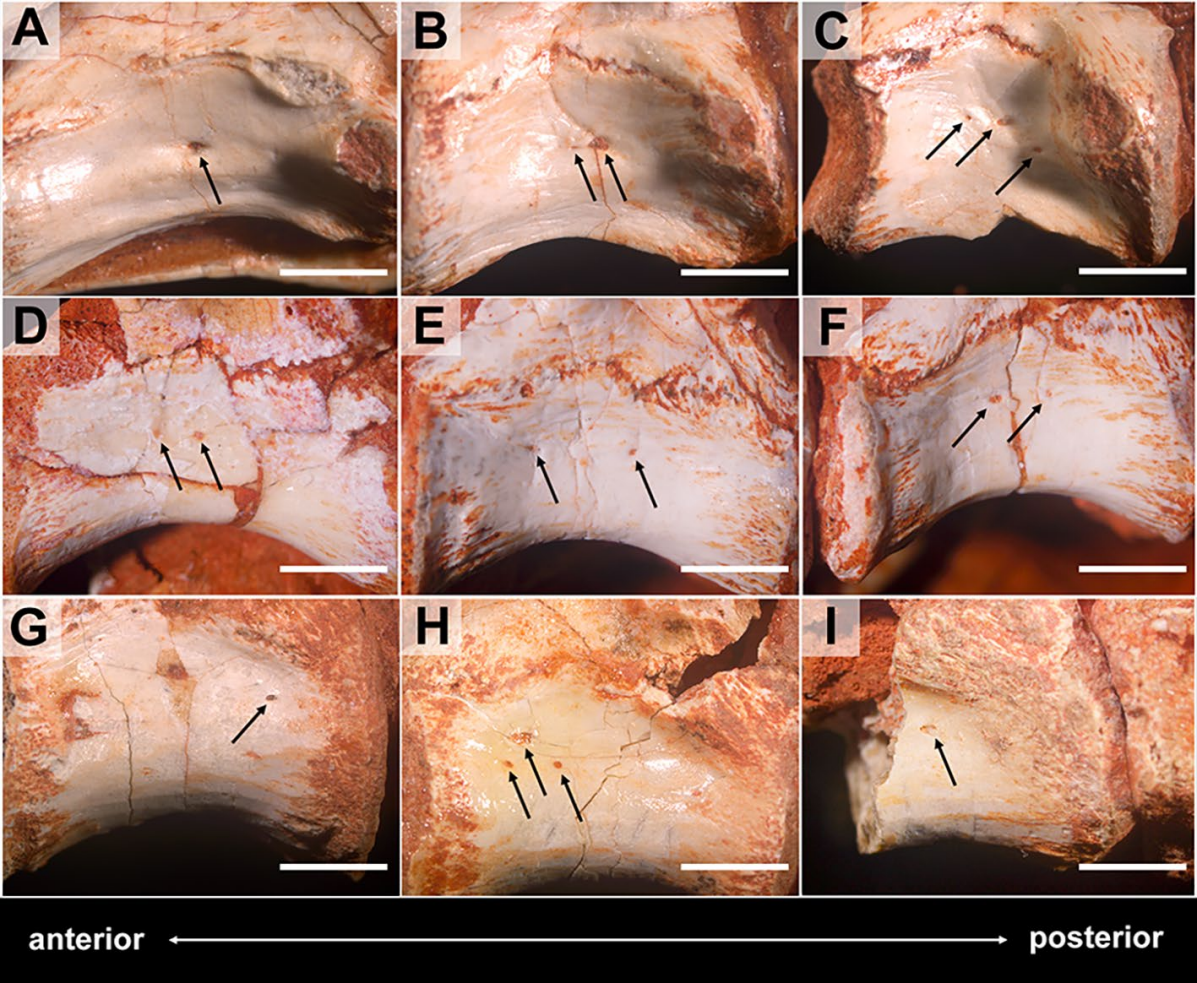
Detail of the vertebrae and foramina of the basalmost sauropodomorph *Buriolestes* (CAPPA/UFSM-0035). Cervical (**A**–**C**), anterior (**D**–**F**) and posterior (**G**–**I**) dorsal vertebrae in right lateral view. Note that nutritional foramina are present throughout the axial skeleton (dark arrows). Anterior/posterior orientation was defined based on the axial position, not the anatomical plane. Scale bar = 5 mm. Figures were generated with Adobe Photoshop CC version 22.5.1 X64.

**Figure 3:**
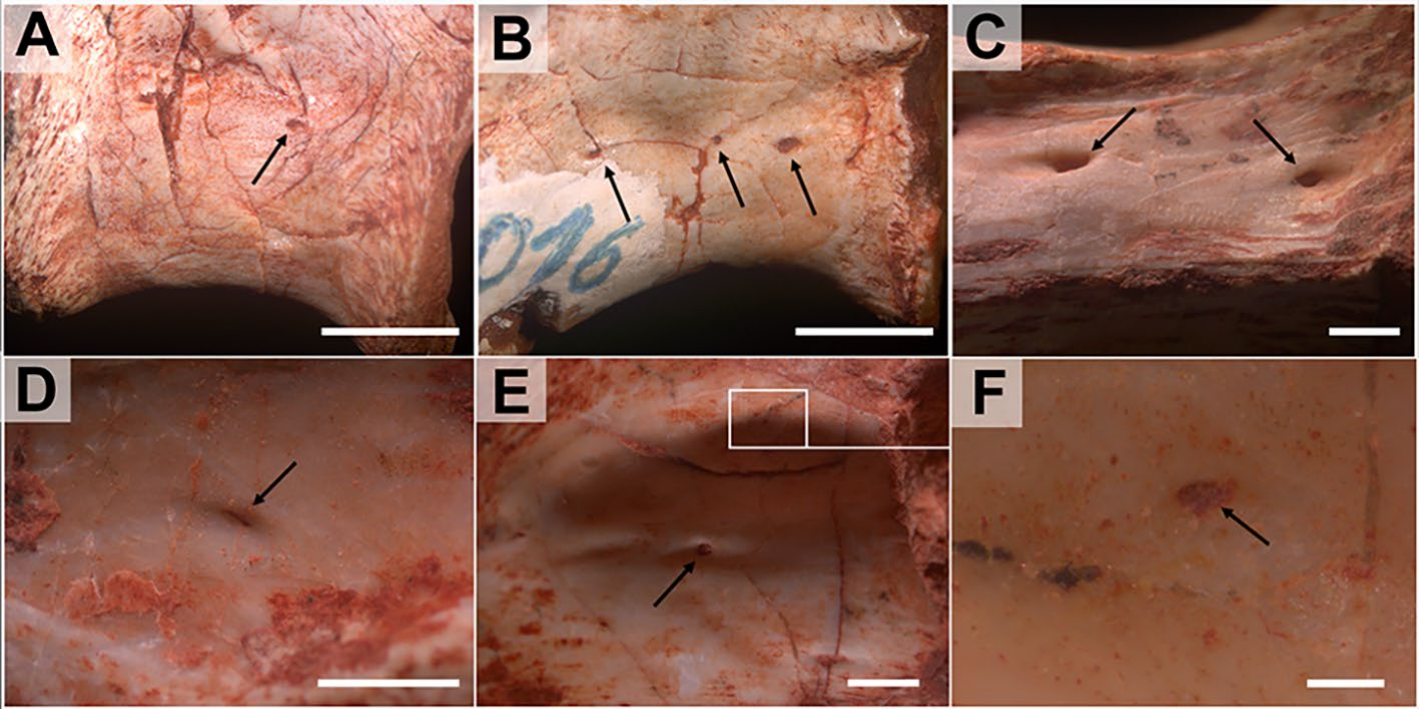
Detail of the vertebrae and foramina of the early sauropodomorph *Pampadromaeus* (ULBRA-PV016; (**A**,**B**)) and the herrerasaurid *Gnathovorax* (CAPPA/UFSM-0009; (**C**–**F**)). Anterior dorsal vertebrae in (**A**,**B**). Anterior cervical in **C** and posterior cervical in (**D**–**F**). Nutritional foramina are present on the lateral surface of the centra (small arrows) in (**A**,**B**) and (**D**–**F**. They present a diminutive diameter when located inside the lateral fossae (**D**–**F**) and are broader on the ventral portion of the centra (**C**). Scale bar in (**A**,**B**) = 5 mm; in (**C**–**E**) = 1 mm; in (**F**) = 0.1 mm. Figures were generated with Adobe Photoshop CC version 22.5.1 X64.

The neural arches of the three taxa show laminae and fossae with moderate complexity compared to what is observed in derived saurischians. *Gnathovorax* presents more robust vertebrae with deeper lateral fossae at the centra than the sauropodomorphs. *Buriolestes* and *Pampadromaeus* vertebrae are rather laterally shorter in anterior view with very subtle lateral fossae in the centra (if any). Pneumatic foramina are absent. However, tiny nutritional foramina abound in almost every element and vary little throughout the vertebral series (Figs. 2, 3). These foramina appear singly, in pairs, and in trios, with no discernible patterns. Lateral foramina present a much greater variance in diameter (0.01–0.9 mm) than the ones occurring ventrally (0.3–0.5 mm). This contrast in size reaches an extreme in the cervicals of *Gnathovorax*, where the ventral foramina (Fig. 3C) are up to ten times broader than the ones occurring inside the lateral fossa (Fig. 3E,F). See the Supplementary Materials for measurements.

**Figure 4:**
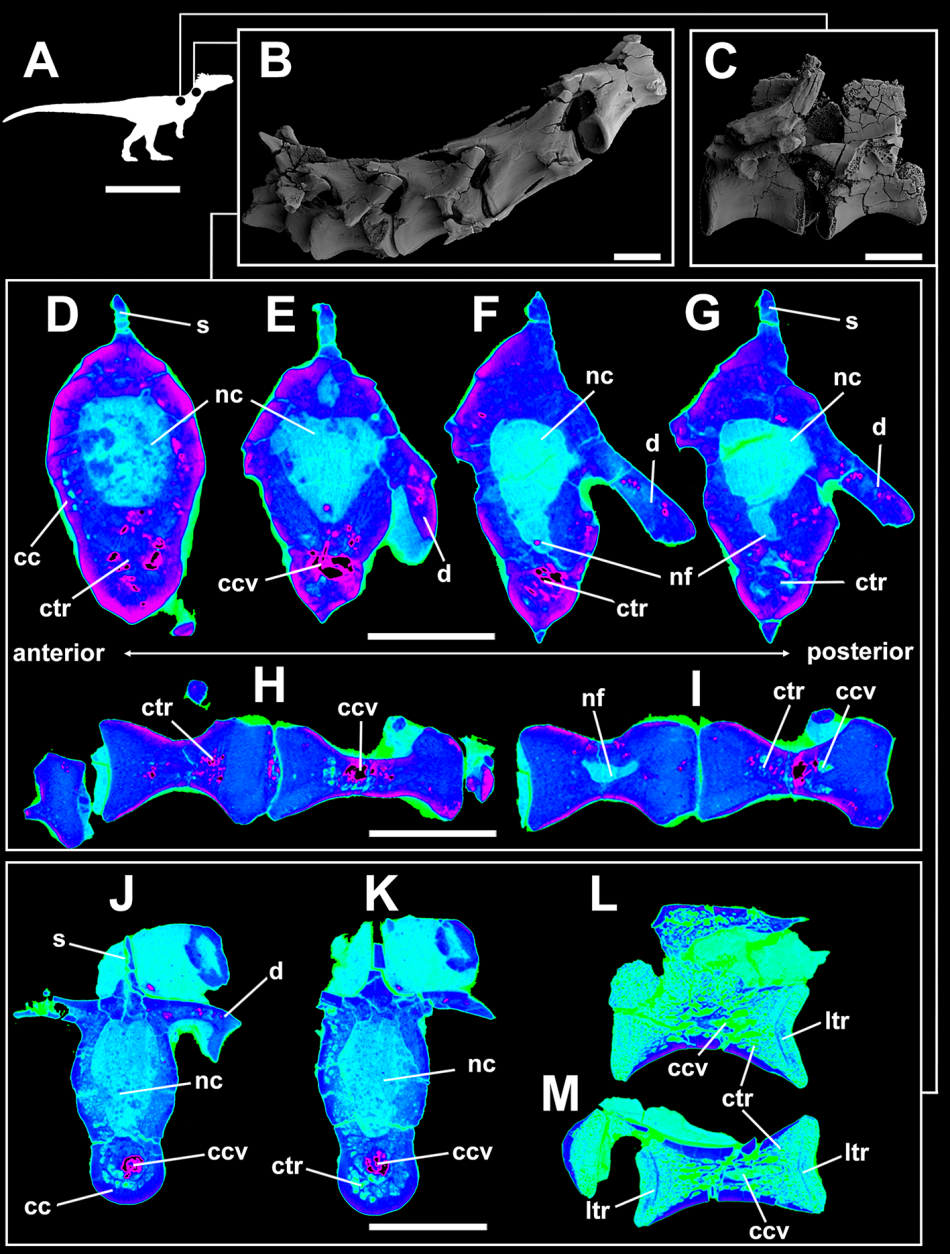
Micro-computed tomography of the vertebrae of the basalmost sauropodomorph *Buriolestes* (CAPPA/UFSM 0035). (**A**) silhouette shows the position of the axial elements. Artist: Felipe Elias. (**B**), three-dimensional reconstruction of the articulated cervical vertebral series and the correspondent high-contrast density slices in (**D–I**). Diagenetic processes partially compromised the internal structures in these cervicals. (**C**), 3D reconstruction of the articulated anterior dorsal vertebrae and the correspondent high-contrast density slices in (**J–M**). Small circumferential chambers occur both ventrally in the dorsal centrum (**J**) and laterally in the neural arch pedicles (**D**). All images indicate apneumatic chaotic trabeculae architecture. Some of the latter develop into larger chambers in the centrum (**E**,**J**,**K**). Nutritional foramina are broader at the bottom of the neural canal in the posterior cervicals (**F**,**G**). All slices were taken from the approximate midshaft. Anterior views in (**D–H**,**J**,**K**). Lateral view in (**L**). Ventral view in (**H**,**I**,**M**). Anterior/posterior orientation was defined based on the axial position, not the anatomical plane. *cc* circumferential chamber, *ccv* chamber in the centrum, *ctr* chaotic trabecula, *d* diapophysis, *ltr* layered trabeculae, *nc* neural canal, *nf* nutritional foramen, *s* neural spine. Scale bar in (**A**) = 500 mm; in (**B–M**) = 10 mm. Computed tomography data processed with 3D Slicer version 4.10. Figures were generated with Adobe Photoshop CC version 22.5.1 X64.

**Figure 5:**
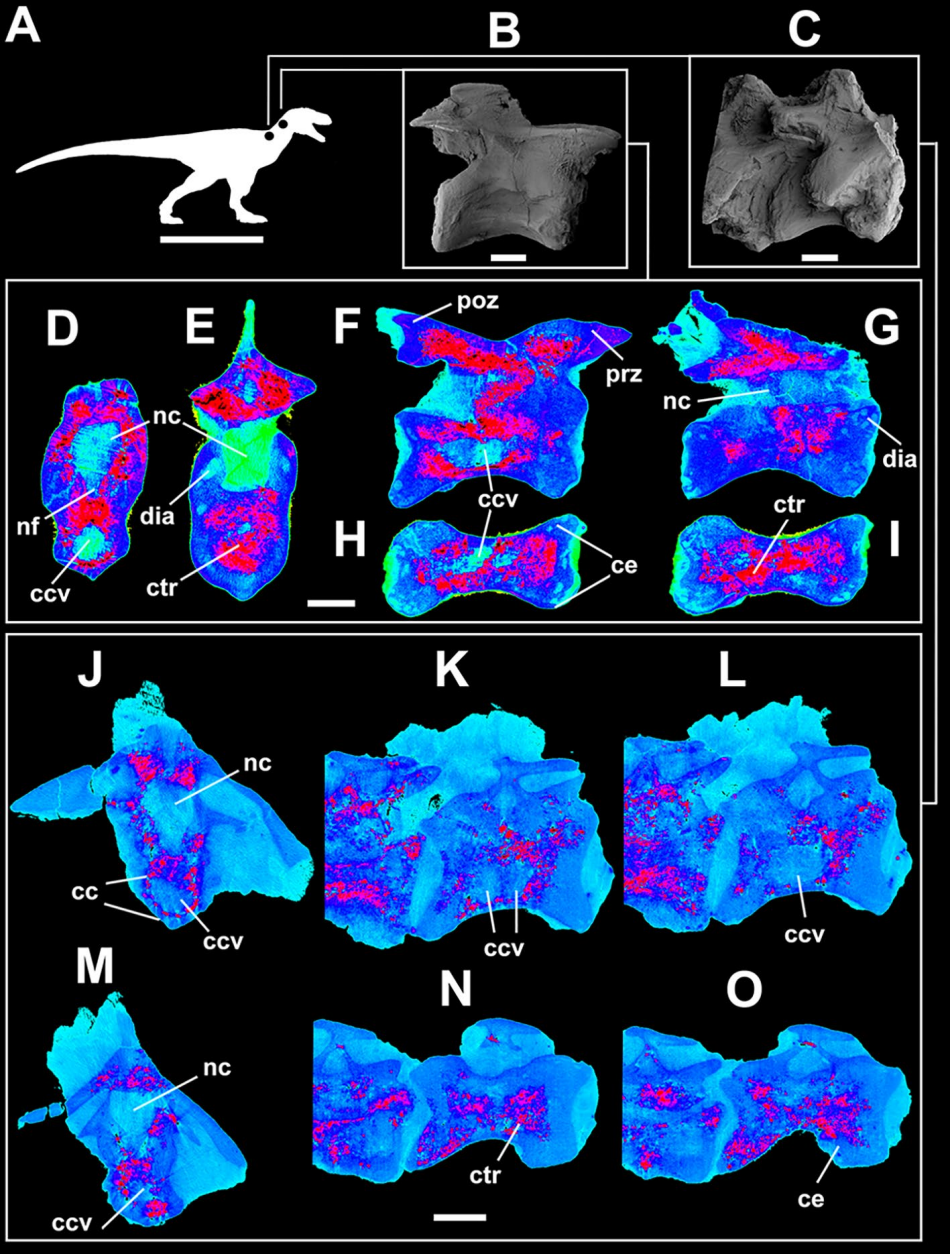
Micro-computed tomography of the vertebrae of the herrerasaurid *Gnathovorax* (CAPPA/UFSM-0009). (**A**) silhouette shows the position of the axial elements. Artist: Felipe Elias. (**B**) 3D reconstruction of the anterior cervical vertebra and the correspondent high-contrast density slices in (**D-I**). Diagenetic artifacts greatly compromised the internal structures. (**C**) 3D reconstruction of the articulated posterior cervical vertebrae and the correspondent high-contrast density slices in (**J–O**). Minerals infilled between trabecular vacancies generate reddish anomalies. All images indicate irregular, chaotic, apneumatic architecture. Note the apneumatic large chambers in the centrum (ccv) and the smaller circumferential chambers at the bottom (cc). All slices were taken from the approximate midshaft. Anterior views in (**D**,**H**,**I**). Right lateral view in (**E**,**L**,**M**). Ventral view in (**F**,**G**,**J**,**K**). *cc* circumferential chambers, *ccv* chamber in the centrum, *ce* centrum, *ctr* chaotic trabeculae, *d* diapophysis, *dia* diagenetic artifact, *nc* neural canal, *nf* nutritional foramen, *poz* postzygapophysis, *prz* prezygapophysis. Scale bar in (**A**) = 1000 mm; in (**B**–**O**) = 10 mm. Computed tomography data processed with 3D Slicer version 4.10. Figures were generated with Adobe Photoshop CC version 22.5.1 X64.

**Figure 6:**
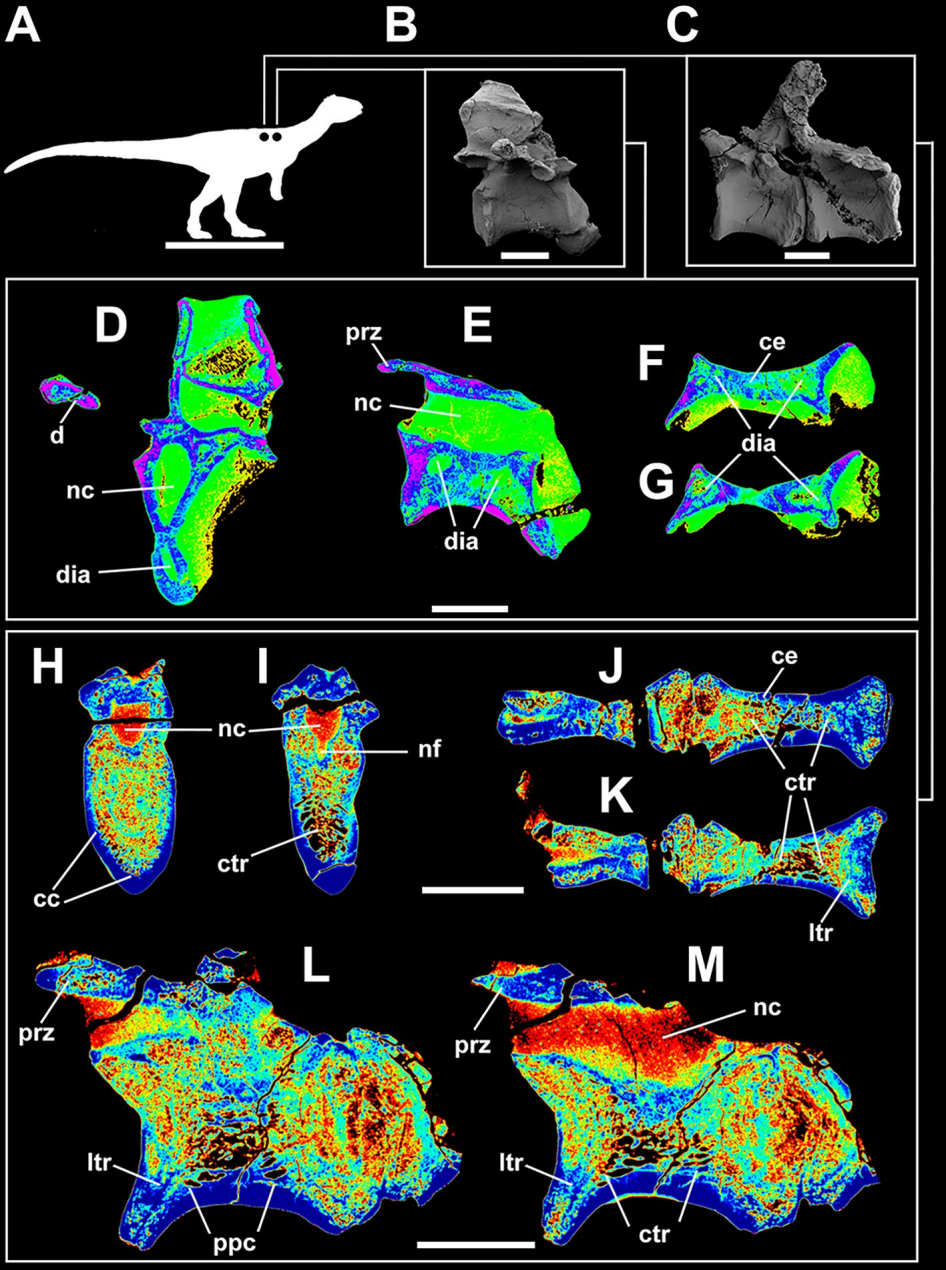
Micro-computed tomography of the vertebrae of the early sauropodomorph *Pampadromaeus* (ULBRA-PV016). (**A**) silhouette shows the position of the axial elements. Artist: Felipe Elias. (**B**) 3D reconstruction of the mid-dorsal vertebra and the correspondent high-contrast density slices in (**D**–**G**). Diagenetic artifacts significantly compromised the internal structures in this element. (**C**) 3D reconstruction of the articulated anterior dorsal vertebrae and the correspondent high-contrast density slices in (**H**–**M**). Note the circumferential chambers at the bottom of the centrum in (**H**). Also, note the ‘pseudopolycamerate’ architecture in (**L**). All images indicate irregular, chaotic, apneumatic architecture. All slices were taken from the approximate midshaft. Anterior views in (**D**,**H**,**I**). Right lateral view in (**E**,**L**,**M**). Ventral view in (**F**,**G**,**J**,**K**). *cc* circumferential chambers, *ce* centrum, *ctr* chaotic trabeculae, *d* diapophysis, *dia* diagenetic artifact, *ltr* layered trabeculae, *nc* neural canal, *nf* nutritional foramen, *ppc* ‘pseudopolycamerate’ architecture, *prz* prezygapophysis. Scale bar in (**A**) = 500 mm; in (**B**–**M**) = 10 mm. Computed tomography data processed with 3D Slicer version 4.10. Figures were generated with Adobe Photoshop CC version 22.5.1 X64.

**Figure 7:**
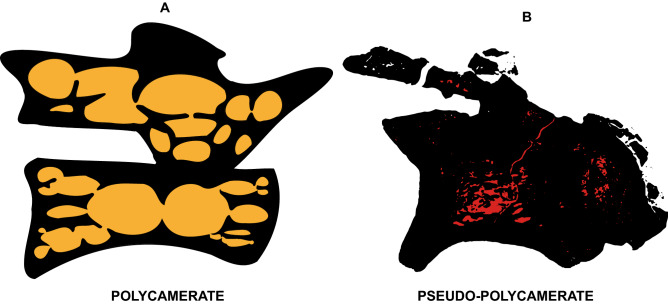
Schematic profile of the internal architecture of two saurischians. (**A**) *Dilophosaurus*, showing a polycamerate pneumatic structure. (**B**) *Pampadromaeus*, demonstrating pseudo-polycamerate apneumatic trabeculae. (**A**) is based on Marsh^38^ and Brum et al.^36^. Not to scale. Figures were generated with Adobe Illustrator CC version 20 X64.

Taphonomy greatly affected some vertebrae and, consequently, influenced some μCT scan slices. Sometimes such differences occur within the same specimen (as in *Buriolestes* and *Pampadromaeus*; Figs. 4, 6). Nonetheless, the data obtained from this analysis successfully provide information on the internal architecture of the vertebrae in all three taxa (Figs. 4, 5, 6). The neural arches feature a homogenous texture of dense trabeculae in all specimens, and the greatest structural diversity is located inside the centra. These comprise mostly apneumatic chaotic trabeculae (ctr; Figs. 4, 5, 6). There are non-pneumatic chambers (ccv) inside certain elements of *Buriolestes* (Fig. 4E,H–M) and *Gnathovorax* (Fig. 5D,F,H,J–M). Small circumferential chambers (cc) populate the centra dorsally in all specimens (Fig. 4J; Fig. 5D,J,M; Fig. 6H) and also laterally in the pedicles in *Buriolestes* (Fig. 4D). Two layered trabeculae (ltr) are present in the cotyles of both sauropodomorphs but not in the herrerasaurid. *Pampadromaeus* features a ‘pseudo-polycamerate’ (ppc) architecture (Fig. 6L). Here we define ‘pseudo-polycamerate’ as chaotic apneumatic trabecular chambers infilled with blood and fat tissues but resemble the fractals of the pneumatic polycamerate as defined by Wedel^29^. Figure 7 illustrates both architectures side-by-side in high-contrast monochromatic vertebral profiles. Something similar exists in the earlier *Buriolestes* but the configuration, in this case, is rather a combination of chaotic trabeculae and elongated chambers (Fig. 3L,M). Finally, the neural canal nutritional foramina are broader towards the dorsal-cervical joint, as shown in *Buriolestes* and *Gnathovorax* (Fig. 4D–G and Fig. 5D,J,M). These large vascular foramina in the floor of the neural canal were probably associated with the basivertebral venous system^39,40^.

## Discussion

The microtomography of the three specimens evidences the absence of unambiguous PSP. Our data also support that a better description of the foramen anatomy can solve questions on ambiguous PSP^41^. Tiny foramina (< 1 mm) inside weakly developed fossae are vascular in origin instead of indicative of pneumatization^3,41^. Proportionally large-diameter foramina (e.g., > 5 mm, in our specimens) associated with deep fossae must be taken into consideration when diagnosing unambiguous PSP^3,41^. Those latter must also be connected with clear internal pneumatic architecture^3,30,41^. Most pneumatic vertebrae are internally organized into camerate and camellate bone tissues, and these are the best macroscopic correlates of bone-penetrating pneumatic diverticula^11,13,29,36,37,42,43^. These Carnian taxa failed to show an architecture consistent with pneumatization, such as that observed in the derived sauropods *Saltasaurus* (PVL 4017–17/214)^11^ and *Ibirania* (LPP-PV-0200)^37^, and theropods like *Aoniraptor* (MPCA-Pv 804/1-25)^43^ and noasaurids (DGM 929-R)^36^. Even giant basal neosauropods such as *Apatosaurus* (OMNH 01094)^10^ and *Giraffatitan* (MB.R. 2180.25/26)^44^ present large organized cortical-tissue camerae suggesting interaction with pneumatic diverticula. Lastly, our data indicate that an invasive pneumatic system was not present in the postcranial skeletons of the earliest Carnian dinosaurs. The earliest evidence of PSP occurred in the fossae adjacent to the diapophysis in most basal sauropodomorphs^45^. Our scans show that in *Buriolestes*, *Pampadromaeus*, and *Gnathovorax* any fossae associated with the neural arches or diapophyses are shallow, simple (i.e., not divided into subfossae sensu Wilson^27^), and do not represent invasive pneumatization of the vertebrae.

**Figure 8:**
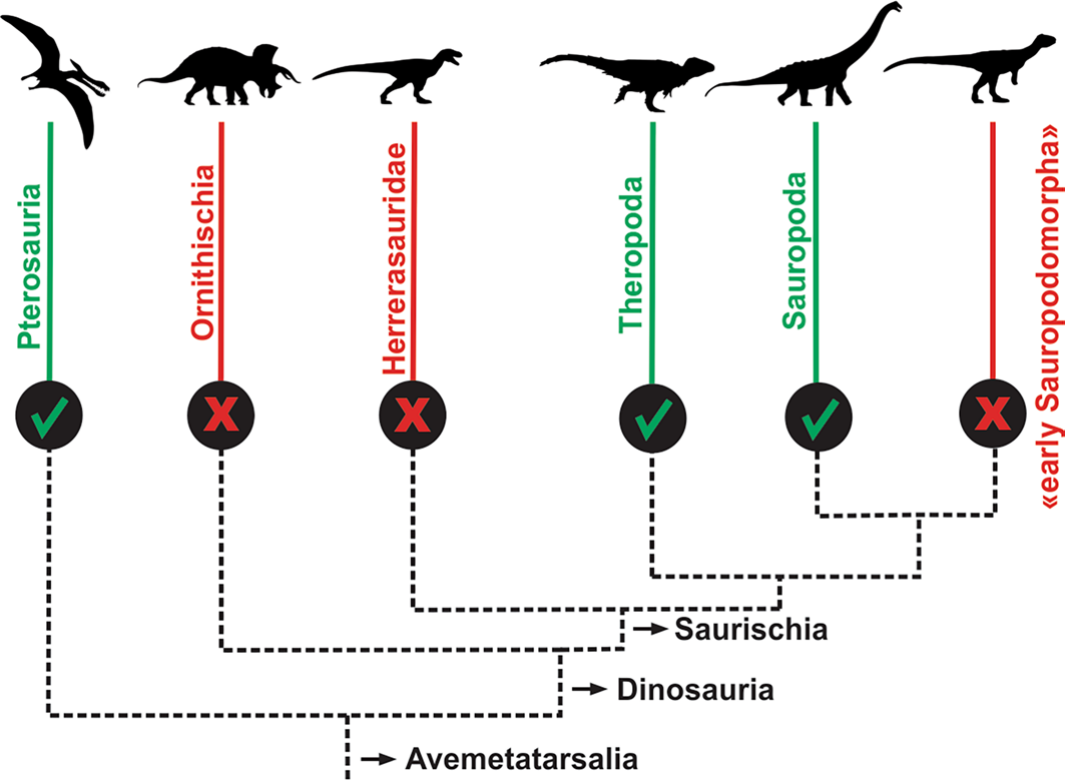
A simplified cladogram of Avemetatarsalia illustrates the branches in which the unambiguous presence of an air sacs system has been evidenced (bright/green ‘check’ sign). The absence of an air sacs system in the oldest dinosaurs presented in this study eliminates the hypothesis of the homology of this trait between pterosaurs and saurischians. Our results also corroborate that this trait appeared independently in three clades: Pterosauria, Theropoda, and Sauropoda. “Early Sauropodomorpha” is a paraphyletic branch. Cladogram based on Novas et al.^54^. Silhouettes authored by Felipe Elias, Scott Hartman, Tasman Dixon, Jagged Fang, and FunkMonk. Figures were generated with Corel Draw X6 version.

The evidence of the absence of PSP in the earliest dinosaurs suggests that invasive PSP found in theropods, sauropods and pterosaurs was not homologous^3,15,30,46,47^. This is solid evidence that invasive intraosseous pneumatization must have evolved at least three times independently (see Fig. 8). Our results are also in accordance with recent findings that demonstrate the nonexistence of PSP in the early ornithischian *Heterodontosaurus*^47^*.* Nonetheless, this still does not exclude the hypothesis indicating that the homology of the underlying non-invasive pulmonary tissue could be an ancestral ornithodiran condition^15,48^. In extant birds, many diverticula do not interact with the skeleton, including intermuscular, intervisceral, and subcutaneous diverticula^49^. However, similar ‘cryptic’ diverticula^50^ in basal ornithodirans would be unlikely to preserve in the Triassic fossil record, and by definition, such diverticula do not leave diagnostic skeletal traces, and both of these factors complicate any attempts to investigate the earliest stages in the evolution of PSP. Even histological evidence of the respiratory diverticula requires interaction with the bone to survive fossilization^4,33,37,43,51^.

Some early non-archosaurian archosauriforms such as *Erythrosuchus* present developed fossae, laminae, and even foramina which led some authors to propose ancestral evidence of PSP^52^. However, we suggest a careful analysis of foramina size and position, as well as a detailed description of the internal trabecular architecture. These are the definitive macroscopic indicators of unambiguous PSP, as previously hypothesized^3,30,41^. *Erythrosuchus* microCT scan (NHMUK R3592)^15^ revealed a chaotic array of trabecular walls in a very dense, near-granular, mass of thick bone throughout the vertebra (Fig. 6 in^15^). Additionally, the absence of connections between internal structures with external foramina corroborates with the apneumatic status of *Erythrosuchus*^15^.

The short time and phylogenetic distances between the early sauropodomorph *Buriolestes* and the later *Pampadromaeus* allowed the observation of a short step in the evolution of vertebral vasculature. The increasingly irrigated internal architecture with chaotic trabecular vacancies and chambers in basal dinosaurs, filled with blood and fat tissues, could have favored the evolution of the true pneumatic structures in the latest Triassic. *Pampadromaeus* was excavated in a stratum slightly younger than *Buriolestes* and presented a pseudo-polycamerate vascularized architecture, much different from the chaotically organized pattern of the older *Buriolestes*. This change demonstrates the increasing complexity of these tissues that may have laid the groundwork for the later origin of true unambiguous PSP.

We note many suggestive correspondences between the apneumatic vertebral internal structure of these Carnian dinosaurs and the pneumatic internal structures of more derived saurischians. Similar apneumatic chambers were also described in the vertebral centra of non-avemetarsalian archosauromorphs such as in the centra of the *Alligator* (see Fig. 1C,D) and in the neural arches of taxa such as *Erythrosuchus*^15^*.* In early saurischians, in addition to the pseudo-polycamerate architecture discussed above, apneumatic chambers in the centra (ccv) of the Carnian taxa are structurally similar to pneumatic camerae of later sauropods and theropods (Fig. 6)^29^. Even more compellingly, small circumferential chambers (cc) and layered trabeculae (ltr) in these early dinosaurs resemble circumferential pneumatic chambers and radial camellae in derived titanosaurian sauropods^37^. In extant birds, developing pneumatic diverticula have been observed to follow pre-existing blood vessels^3,53^. If pneumatization followed vascular pathways in extinct dinosaurs^41^, that could explain why pneumatic internal structures in later, more derived dinosaurs resemble the ancestral, apneumatic structures documented in this study. A detailed study of the ontogenetic development of vertebral pneumaticity in a derived sauropod or non-avian theropod, using CT and bone histology, is needed to elucidate the relationship between pneumatic internal structures and the apneumatic structures that predate them (ontogenetically and phylogenetically).

Eventually, the analysis of Norian bagualosaurian taxa will have the potential to provide evidence of the rise of unambiguous PSP, and the appearance of the invasive pneumatic system that we observe in later avemetatarsalians, including extant birds.

## Conclusions

We analyzed three of the earliest dinosaurs and our data fill a gap in the knowledge of the evolution of the Respiratory System. Selected highlights are listed below:

- Microtomography of the three specimens indicates the absence of unambiguous postcranial pneumaticity. Therefore, we conclude that an air sacs system permeating the skeleton was not present in the earliest dinosaurs (late Carnian).
- The evidence of the absence of PSP in the earliest saurischian dinosaurs corroborates the hypothesis that the invasive air sac systems found in theropods, sauropods and pterosaurs were not homologous. This is solid evidence that unambiguous PSP evolved at least three times independently in those clades. Nonetheless, this still does not exclude the other hypothesis indicating that the homology of the underlying pulmonary tissue could be an ancestral ornithodiran condition.
- The chaotic organization of the larger internal trabecular vacancies and chambers, originally filled with blood and fat tissues, could have favored the evolution of the true pneumatic structures in the latest Triassic. *Pampadromaeus* was slightly younger than *Buriolestes* and already presented a pseudo-polycamerate structure, supporting the increasing complexity of these tissues that would later favor the origin of true unambiguous PSP.

Finally, investigations regarding Norian taxa are crucial in order to provide evidence on the rise of unambiguous PSP, and the appearance of the air sacs system as we know it today from the surviving avemetatarsalians (see Table S1 in supplementary materials).

## Supplementary Information

Supplementary Tables.

## Data Availability

All fossils are housed in a public research institution and can be accessed with a request to the curator of the collection. Microtomography data are available upon request by email to the corresponding author.
